# Contrasting genetic diversity and ecological niche modelling of the montane grass mouse *Akodon montensis* in the south of the Atlantic Forest

**DOI:** 10.1098/rsos.251629

**Published:** 2025-11-12

**Authors:** Carolina Alicia Labaroni, Andrea P Tarquino-Carbonell, Noelia Soledad Vera, Rosio Gabriela Schneider, Leandro Maciel Buschiazzo, Romina Vanessa De Cena, Gabriela García, Marina Beatriz Chiappero, Dardo Andrea Marti, Cecilia Lanzone

**Affiliations:** ^1^ Laboratorio de Genética Evolutiva, Facultad de Ciencias Exactas Químicas y Naturales, Instituto de Biología Subtropical – Nodo Posadas (CONICET-UNaM), Posadas, Misiones Province, Argentina; ^2^ Instituto Argentino de Investigaciones de las Zonas Áridas, Mendoza, Mendoza Province, Argentina; ^3^ Cátedra de Genética de Poblaciones y Evolución, Universidad Nacional de Córdoba, Facultad de Ciencias Exactas Físicas y Naturales, Córdoba Province, Argentina; ^4^ Instituto de Diversidad y Evolución Austral (IDEAUS-CONICET), Puerto Madryn, Chubut, Argentina; ^5^ Instituto de Diversidad y Ecología Animal, CONICET and Universidad Nacional de Córdoba, Córdoba Province, Argentina

**Keywords:** biodiversity hotspot, cytochrome b, demographic history, microsatellite, phylogeography, rodent

## Abstract

*Akodon montensis* is widely distributed throughout the Atlantic Forest (AF) hotspot biodiversity, encompassing Brazil and reaching its southern limit in eastern Paraguay and northeastern Argentina. Here, we combined analysis of molecular data and ecological niche modelling to contribute to elucidating its evolutionary history. At a local scale, we studied the genetic variability in microsatellite loci in populations from the remaining AF in Misiones province, Argentina. The moderate genetic differentiation observed in some populations suggests that limited gene flow may result from habitat fragmentation at the south of the AF. At a wide geographic range, the ecological niche modelling identified areas of high environmental suitability for *A. montensis* during the last glacial maximum (LGM) on the coast of Brazil, where the forested habitats expanded onto the continental shelf. This could explain the high diversity in the cytochrome b in this region and contiguous areas, agreeing with the Atlantis Forest hypothesis. Additionally, we observed an extended area of high habitat suitability during the LGM and at present in southwestern Brazil, eastern Paraguay and northeastern Argentina. The evolutionary history of *A. montensis* seems to have been influenced by demographic processes that occurred at different times and regions, shaping its genetic variability and structure.

## 1. Introduction

Environmental and landscape changes that occurred throughout the evolutionary history of taxa have repeatedly altered the dynamics and structure of their natural populations [1,2]. The expansions or contractions of the species’ ranges are frequently associated with these changes over time. In this sense, several studies have demonstrated the role of the demographic histories of species on the distribution of their genetic variability [2,3]. Small populations that live in fragmented habitats may remain isolated from each other. This would result in reduced gene flow and a loss of genetic variability, affecting the ability to cope with environmental changes and the probability of species persistence [4,5].

One of the biomes that has experienced significant landscape transformations is the Atlantic Forest (AF), which extends along the Atlantic coast of southeastern Brazil, entering inland South America to eastern Paraguay and northeastern Argentina, and comprises a complex of 15 distinct ecoregions [6]. Despite its global recognition as a biodiversity hotspot due to its exceptionally high species diversity and endemism, the AF is currently one of the most threatened and heavily fragmented biomes, with only 11.7% (which represents ~16 377 472 ha) of its pristine vegetation cover remaining [6]. This drastic reduction is principally the result of recent anthropogenic activity, particularly habitat destruction driven by agricultural expansion, forest plantations, urbanization, and related land-use changes [6,7]. The most preserved remnants of AF are found in Misiones province (Argentina), known as the Alto Paraná Atlantic Forest ecoregion [6].

Besides, the structure and composition of the AF have been shaped by older historical processes that occurred at different times. Ancient climatic fluctuations, such as those that occurred during the Quaternary, have influenced the distribution of its biological diversity. Different hypotheses have been suggested in an attempt to explain these processes. One of them proposes that in the Pleistocene, during the last glacial maximum (LGM), the AF retracted. This gave rise to the formation of some isolated, but stable refugial areas, in the north and central region of this biome, where the biota persisted. Following this period, when environmental conditions became favourable, the species colonized different regions from these refuges and expanded to their current distribution. This model, known as Pleistocene Refugia, predicts high species endemism and genetic diversity in refugial zones, but lower endemism and lower genetic diversity with molecular signatures of range expansion after the LGM within species in unstable regions outside the refugia [8]. The results of different phylogeographic studies focused on the central and northern AF are congruent with this hypothesis [9]. However, limited attention has been given to southern AF areas, making the exact location and number of these refuges controversial [10–16]. On the other hand, an alternative hypothesis, known as the Atlantis Forest, proposes that the AF probably expanded, rather than contracted, during the LGM by the emergence of the Brazilian continental shelf. This portion of land, currently submerged, would have played an important role in the evolution of the biodiversity of AF during that period [17]. If the AF did not contract, high genetic diversity indices and absence of expansion signals are expected, at least in the coastal part of its distribution.

The montane grass mouse (*Akodon montensis*, Thomas 1913) is an abundant rodent species that inhabits an important extension of the AF, with a distribution that comprises a large area in southern Brazil, eastern Paraguay and northeastern Argentina [18]. This species occurs in a few northeast provinces of Argentina, mainly in Misiones, that represents its southwestern boundary of distribution [19], and is a natural host of different orthohantaviruses [20, and the references cited therein]. Cytogenetic studies reveal an extraordinary chromosome diversity for the species [21, and the references cited therein]. The first inferences about the molecular diversity of *A. montensis* was a pioneer in the study of its phylogeography, but only a limited number of specimens in a restricted geographic area were analysed [22]. Later, a study with a broader geographic coverage, with specimens mainly from Brazil and Paraguay and a few from Argentina, was presented [14]. This work showed a distribution pattern of genetic variability, and a population history of *A. montensis*, consistent with the Pleistocene Refugia model. In this sense, evidence of high genetic diversity in areas of predicted stability, and molecular signatures of recent range expansion in unstable, recently recolonized regions, were found. However, this expansion was not dated, so it remains unclear whether it took place after the LGM as the model predicts. Additionally, it was suggested the occurrence of three major refuges for *A. montensis:* Bahía, São Paulo (SPR refugium), and Rio Grande do Sul (RGS refugium). This last was proposed for the first time for the southern AF, based on molecular data [14]. Nevertheless, the authors emphasized the need to incorporate additional genetic sampling and paleomodels to clarify the location and extension of the RGS refugium.

The present study aims to evaluate the historical processes that shape the genetic variability and population structure of *A. montensis*. We employed both nuclear and mitochondrial markers for this purpose. We assessed genetic variability at microsatellite loci within a geographically restricted region at the southernmost limit of the species, to detect shorter-term processes such as the contemporary impacts of habitat fragmentation on population structure and to infer more recent demographic events. In order to detect signatures of historical demographic events, and to estimate their temporal framework, we also surveyed the cytochrome b (cyt-b) diversity, a more slowly evolving mitochondrial marker that provides insights into longer-term processes that shape the phylogeographic structure of species. Additionally, we used ecological niche modelling to explore the effects of past and contemporary environmental conditions on genetic patterns throughout the geographic distribution of the species. This integrative approximation could elucidate the evolutionary history of *A. montensis* in a region recognized as an extraordinary biodiversity hotspot.

## 2. Material and methods

### 2.1. Biological sampling, DNA extraction and molecular markers

**Figure 1.**
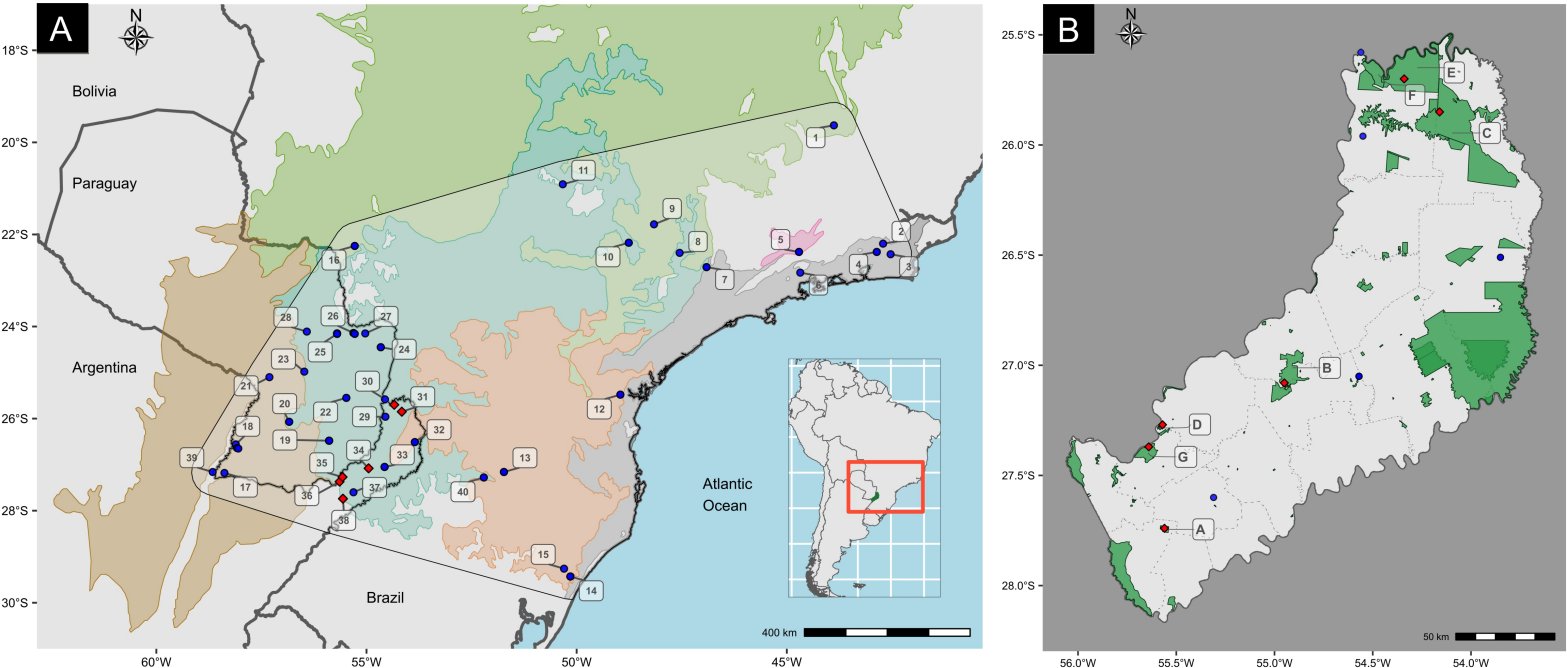
Map showing the sampling localities of *Akodon montensis*. (A) The approximate geographic distribution is marked with a black line and transparent background. Ecoregions are indicated in different colours: Alto Parana Atlantic Forest in light blue, Cerrado in green, Araucaria moist forests light orange, Campos rupestres montane savanna in pink, Serra do Mar coastal forest in grey and Humid Chaco in brown (*Source*: WWF—https://databasin.org/datasets/68635d7c77f1475f9b6c1d1dbe0a4c4c/). The cytochrome b molecular data included in our analyses come from localities 1 to 39. The blue circles correspond to cytochrome b sequences published by Valdez & D’Elía [14], while the red squares represent those obtained in this study. Locality 30 has a circle and a square. The molecular data included in statistical analyses with microsatellite loci come from localities 30, 31, 34, 35 and 38. Localities 1 to 40 were used for ecological niche analysis. (B) Reserves or natural parks in Misiones province sampled in this work: A) Parque Provincial de las Sierras ‘Ing. Raúl Martínez Crovetto’; B) Parque Provincial Salto Encantado del Valle del Arroyo Cuña Pirú; C) Parque Provincial Urugua-í; D) Reserva Privada Osununú; E) Reserva Nacional Iguazú; F) Parque Nacional Iguazú; and G) Parque Federal Campo San Juan.

Small rodents were collected in Misiones province, Argentina (figure 1). We followed the guidelines of the American Society of Mammalogists for handling animals [23]. We extracted DNA from muscle or liver samples of these animals using a saline extraction protocol [24]. Two types of molecular markers were used: microsatellite loci and cyt-b. A total of 10 microsatellite primer pairs were tested, including 2 species-specific primers [25] and 8 designed for *Akodon azarae* [26] (electronic supplementary material, table S1). Four of these loci were successfully amplified and genotyped using fluorescently labelled forward primers (Akom13, Aaz1, Aaz5 and Aaz8) in 79 individuals from five localities of Misiones (electronic supplementary material, table S2), which in figure 1A correspond to points: 30 (Reserva Nacional Iguazú), and 31 (Parque Provincial Urugua-í) in the north of Misiones; 34 (Parque Provincial Salto Encantado del Valle del Arroyo Cuña Pirú) in the centre; 35 (Reserva Privada Osununú), and 38 (Parque Provincial de las Sierras ‘Ing. Raúl Martínez Crovetto’) in the south of Misiones. The locality Parque Federal Campo San Juan (point 36) was excluded from this analysis due to low sample size (*n* = 4). The molecular size of the amplification products was determined at Macrogen Inc. (Seoul, South Korea). Fragments were scored using the software PeakScanner v. 1.0 .

Also, a 638 bp fragment of the cyt-b gene was amplified by polymerase chain reaction (PCR) in 94 specimens of six localities of Misiones. These localities in figure 1A correspond to points: 30 (Reserva Nacional Iguazú), 31 (Parque Provincial Urugua-í), 34 (Parque Provincial Salto Encantado del Valle del Arroyo Cuña Pirú), 35 (Reserva Privada Osununú), 36 (Parque Federal Campo San Juan) and 38 (Parque Provincial de las Sierras ‘Ing. Raúl Martínez Crovetto’) (electronic supplementary material, table S2). We used the primers named MVZ05 (5′-CGAAGCTTGATATGAAAAACCATCGTTG-3′) and MVZ16 (5′-AAATAGGAARTATCAYTCTGGTTTRAT-3′) [27]. Amplifications were performed with an initial denaturation of 3 min at 95°C, followed by 35 cycles of 45 s of denaturation at 93°C, 1 min of annealing at 50°C, 1 min of extension at 72°C, and a final extension of 7 min at 72°C. PCR products were purified with an Accuprep purification Kit (*Bioneer*) and sent to the DNA sequencing service of Macrogen Inc. All mitochondrial sequences obtained were submitted to the GenBank database under accession number PP715535 to PP715628 (electronic supplementary material, table S2). Sequences of the cyt-b gene were edited using MEGA v. 11 [28]. The alignment was conducted using MAFFT v. 7 [29] under the strategy G-INS-i and default parameters for gaps opening and extension gaps (electronic supplementary material, text S1).

### 2.2. Statistical analysis

#### 2.2.1. Microsatellites

Each population was checked for the presence of null alleles and scoring errors (stuttering or large allele drop outs) with Microchecker [30]. Deviations from Hardy–Weinberg equilibrium per locus and population were tested using exact tests with the Markov chain method (10 000 dememorization steps, 20 batches, 5000 iterations) in GENEPOP v. 4.2 [31]. Genetic variability was measured by the observed number of alleles (Na), number of effective alleles (Ne), number of private alleles (PA), observed heterozygosity (Ho), and expected heterozygosity (He) using Genalex v. 6.5 [32]. The genetic differentiation between populations was evaluated by the index Dest [33] with Genalex v. 6.5. To assess the degree of isolation and/or connectivity between populations, we used the Bayesian approach in Geneland v. 4.0.6 [34] implemented in R [35], which combines genetic and spatial data. The analysis was performed under the correlated allele frequencies and null allele models. In the first step, we estimated the number of genetic clusters (*K*) from 1 to 10 using 1 000 000 MCMC iterations and a thinning interval of 100, in five independent runs. Post-process of samples consisted of a burn-in of 2500. The final run was performed using the same parameters mentioned above, with the most likely number of genetic populations previously inferred, i.e. *K* = 4. Isolation-by-distance was assessed using Mantel tests with 999 permutations in Genalex v. 6.5. We use the previously calculated genetic differentiation (Dest) and the geographical distance (in km) between pairs of populations.

We used the R package VarEff [36] to estimate past changes in effective population size (Ne) from microsatellite loci. VarEff implements an approximate Markov chain Monte Carlo (MCMC) approach that estimates the variation in Ne over time based on motif distance frequencies between alleles. As this approach assumes that population structure is negligible, we performed the estimations for each genetic cluster identified by Geneland. We performed several pilot runs, varying several parameters to adjust their values following the authors’ recommendations: JMAX (the number of changes in Ne past) was tested at 2, 4 and 6; three mutation models were examined: the single-step model, the two-phase model and the geometric model, with the parameter C set at 0.20 for the last two models. Finally, the number of generations since the origin of the population was set at 5000, 10 000 and 20 000, in order to cover several time periods since the end of the last glaciation, conservatively considering two generations per year. The mutation rate was set to 1 × 10^–4^ [37,38], the value of DMAX (the maximum distance between alleles in repeat units) was chosen so that the frequencies of pairs of alleles separated by DMAX or more are below 0.005, and the prior for past Ne was set at Θ_1_/(4 u) [36]. All other parameters were set to default. Analyses were run with 10 000 batches of length 10, thinning of 100 and a burn-in of 10 000. The demographic trajectories were plotted as the harmonic mean of Ne in 100 generations intervals, using the NatSizeDist function in VarEff.

#### 2.2.2. Mitochondrial DNA

##### 2.2.2.1. Genetic diversity

In total, 179 cyt-b partial sequences were analysed (electronic supplementary material, table S2). We analysed 94 cyt-b partial sequences of *A. montensis* obtained in this work from Misiones, Argentina, along with the previously published sequences [14] available in GenBank from Argentina (*n* = 12), Paraguay (*n* = 45), and Brazil (*n* = 28). Different indices of genetic diversity, including number of haplotypes (h), haplotype diversity (Hd), and nucleotide diversity (Pi), were calculated with DnaSP v. 5.10 [39]. Nucleotide differences and genetic distances (using the *p*-distance) for all pairwise comparisons were calculated with MEGA v. 11 [28]. A haplotype network was inferred using the median-joining algorithm implemented in the NETWORK v. 5.0 software [40], and its graphic edition was done with the CorelDraw X7 programme.

##### 2.2.2.2. Demographic and spatial diffusion analyses

We assessed the demographic history of populations using the neutrality tests of Tajima’s *D* and Fu’s Fs [41,42], and tested for recent demographic expansion using mismatch distribution analyses [43]. These analyses were performed with DnaSP v. 5.10 [39]. In addition, we performed a Bayesian skyline plot (BSP) analysis in BEAST v. 2.7.3 [44] to examine changes in population size through time. The analysis was run for 100 million generations and sampled every 1000 steps. For this, we used a substitution rate of 2.3% per million years estimated for the cyt-b gene for South American sigmodontine rodents [27]. We additionally assessed the spatial dynamics of *A. montensis* over time using a lognormal relaxed random walk model implemented in the software BEAST [44]. The analysis was performed including all sequences (*n* = 179) and their respective geographic coordinates. The substitution model implemented was HKY+G, according to the model selection analysis following the Akaike information criterion in jModeltest v. 2.1.3 [45]. We used a normally distributed diffusion rate and a coalescent GMRF Bayesian Skyride Model. The mutation rate was the same as used for the BSP analysis. We set the jitter option to 0.01 to add variation to sequences with the same coordinates. We performed the final run with 300 million generations, sampled at every 30 000 steps. We used Tracer v. 1.7.2 to check the stationarity parameters. Later, we obtained the maximum clade credibility tree using TreeAnnotator v. 1.7.5. This was used as the input file for Spread v. 1.0.7 [46] to reconstruct the pattern of spatial diffusion. The results were visualized in Google Earth Pro.

### 2.3. Ecological niche modelling

The localities used for ecological niche modelling are shown in figure 1. This dataset includes specimens collected in this study and previous works [14,47], all georeferenced under WGS 84. Climatic variables were extracted from WorldClim v. 1.4 (https://www.worldclim.org/) [48]. Models were projected under current and historical climate scenarios. Historical scenarios were generated using global circulation models (GCMs), which simulate past climate conditions through mathematical algorithms [49,50], while current scenarios represent conditions between 1960 and 1990 [48]. We used WorldClim as the baseline for all projections, and historical conditions were modelled for the Mid-Holocene (~6000 years ago) and the LGM (~22 000 years ago), using the same set of bioclimatic variables reflecting annual trends, seasonality and precipitation/temperature extremes [48]. Projections were made at 2.5 arc-minute resolution using two GCMs: CCSM v. 4 and MIROC-ESM [51,52].

We estimated Spearman correlation coefficients (*r*) for climate variables extracted from the occurrence points using the QGIS ‘Point Sampling Tool’ plugin [53]. Highly correlated variables (*r* > 0.8) were excluded to reduce multicollinearity. Correlation analyses were conducted with the Hmisc package in R [35], resulting in the selection of eight bioclimatic variables (electronic supplementary material, material.zip). The study area was defined as the accessible region for the species, based on a buffered minimum convex polygon around occurrence points, generated in QGIS [54].

Ecological niche models were constructed using MaxEnt v. 3.4.1 (https://biodiversityinformatics.amnh.org/open_source/maxent/), which applies the principle of maximum entropy to estimate species distributions [55]. Model projections were visualized in QGIS using suitability thresholds (0–1) represented by colour gradients from white (null) to red (high suitability). Variable contributions were assessed through jackknife tests. Model parameters—feature classes and regularization multipliers—were tuned using the ENMeval package in R [56], across a range of beta multipliers (0.5–4). Models were evaluated using three criteria: omission rate (OR), area under the curve (AUC), and corrected Akaike information criterion (AICc) [55,56]. OR was calculated based on the lowest predicted suitability among the calibration points. Among models with the lowest OR, we selected the one with the highest test AUC [57], while also considering AICc due to known limitations of AUC related to sampling bias. Final models were built using the optimal parameter settings.

## 3. Results

### 3.1. Microsatellites

All loci used in this study were polymorphic (genotypes and geographic coordinates are in the electronic supplementary material, text S2). Departures from Hardy–Weinberg equilibrium (*p* < 0.05) were found in some loci and populations: Aaz1 in Martínez Crovetto and Aaz8 in Iguazú and Martínez Crovetto (electronic supplementary material, table S3). Microchecker analysis identified that these deviations were due to the presence of null alleles. These were detected in locus Aaz1 in Martínez Crovetto (frequency = 0.23) and in locus Aaz8 in Martínez Crovetto (frequency = 0.24) and Iguazú (frequency = 0.11) (electronic supplementary material, table S4).

Genetic diversity parameters for each population (Na, Ne, PA, Ho and He) are shown in table 1. The average observed heterozygosity (Ho) ranged from 0.6 to 0.8. In total, 79 different alleles were detected, with 18 being private for different populations, and most were reported in the populations of northern Misiones: Iguazú (7) and Urugua-í (7) (table 1).

**Table 1.** Genetic diversity measures at microsatellite loci for populations of *Akodon montensis*.

population	N	Na	Ne	PA	Ho	He
Iguazú (30)	17	12.5	8.35	7	0.809	0.869
Urugua-í (31)	25	14.5	8.63	7	0.838	0.865
Cuña Pirú (34)	14	10.5	7.9	1	0.857	0.852
Osununú (35)	16	10	6.98	1	0.806	0.826
Martínez Crovetto (38)	7	6.75	4.7	2	0.643	0.783

The number of individuals analyzed (N), number of different alleles (Na), effective number of alleles (Ne), private alleles (PA), observed heterozygosity (Ho) and expected heterozygosity (He) are indicated. Numbers in parentheses correspond to locality number in figure 1A.

**Figure 2.**
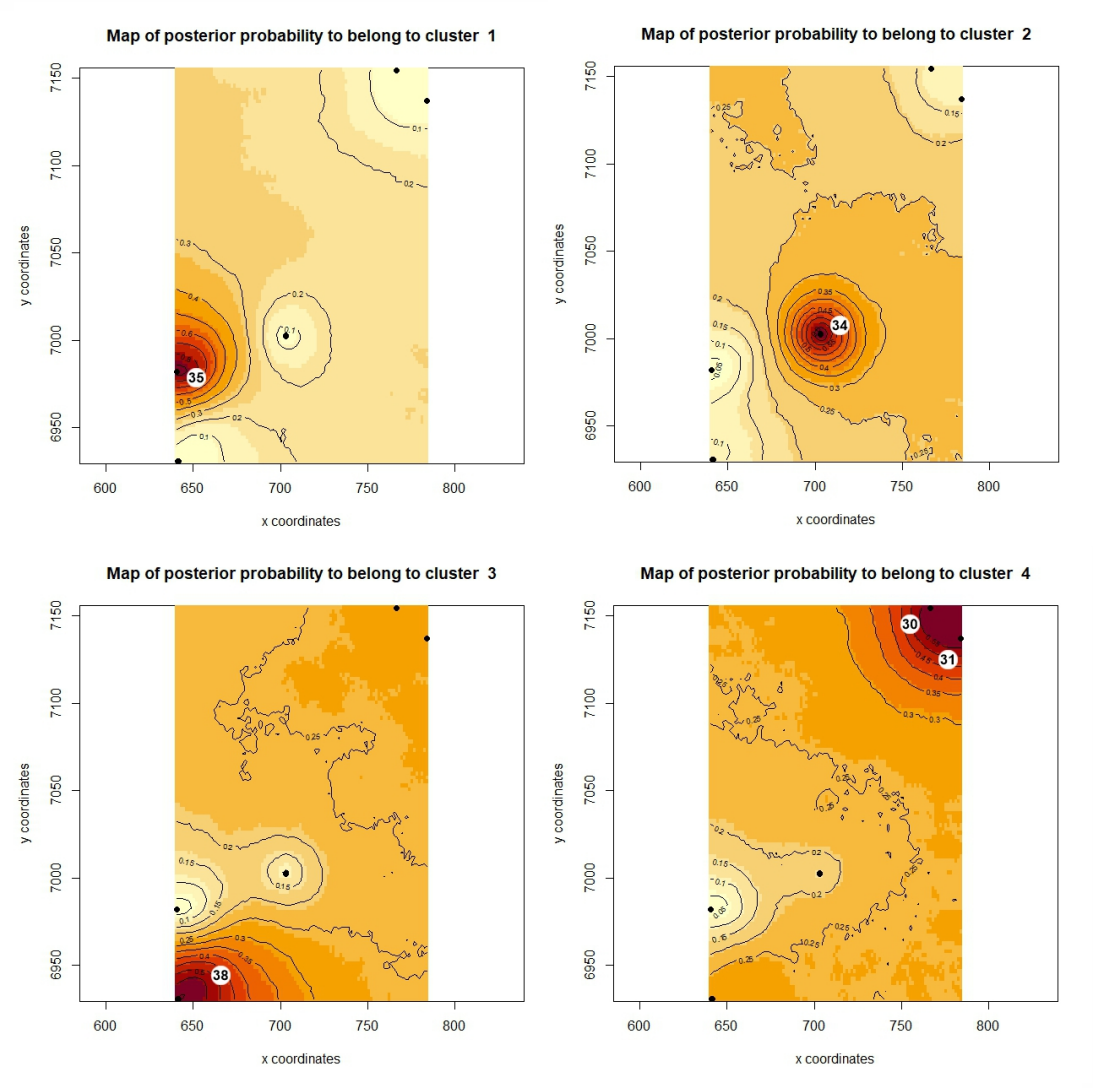
Spatial distribution of the four genetic clusters estimated by Geneland for *Akodon montensis*. Black dots represent groups of individuals. The highest membership values are shown in red. Cluster 1 with individuals from Osununú (35; D) in the south of province; Cluster 2 with individuals from Cuña Pirú (34; B) in the centre of Misiones; Cluster 3 with individuals from Martínez Crovetto (38; A); and Cluster 4 with individuals from Iguazú (30; E) and Urugua-í (31; C) in the north of Misiones. The numbers and letters in parentheses indicate the origin of the samples, as referenced in figure 1A,B, respectively.

The mean Dest value for all loci was 0.09 (*p* = 0.018), showing moderate genetic differentiation between all sampling sites. A high and significant Dest value was found between Cuña Pirú and Martínez Crovetto (Dest = 0.201; *p* = 0.040) and between Cuña Pirú and Osununú (Dest = 0.132; *p* = 0.023; table 2). Geneland identified four genetic clusters (*K* = 4) in the study area. Cluster 1 comprises individuals from Osununú (0.80 of posterior probability, PP). Cluster 2 includes individuals from the central population Cuña Pirú (PP = 0.60). Cluster 3 comprises individuals from Martínez Crovetto (PP = 0.60). Cluster 4 includes individuals of Iguazú and Urugua-í in northern Misiones (PP = 0.55) (figure 2). The Mantel statistic was non-significant, indicating the absence of isolation by distance (*r* = −0.041; *p* = 0.387).

**Table 2.** Pairwise Dest estimates and *p* values between populations of *Akodon montensis*. Statistically significant Dest values are highlighted in bold. Numbers in parentheses correspond to locality number in figure 1A.

region	north		centre	south	
population	Iguazú	Urugua-í	Cuña Pirú	Osununú	Martínez Crovetto
Iguazú (30)	0	0.821	0.184	0.157	0.143
Urugua-í (31)	−0.047	0	0.166	0.25	0.147
Cuña Pirú (34)	0.056	0.057	0	0.023	0.040
Osununú (35)	0.060	0.028	**0.132**	0	0.078
Martínez Crovetto (38)	0.115	0.101	**0.201**	0.160	0

**Figure 3.**
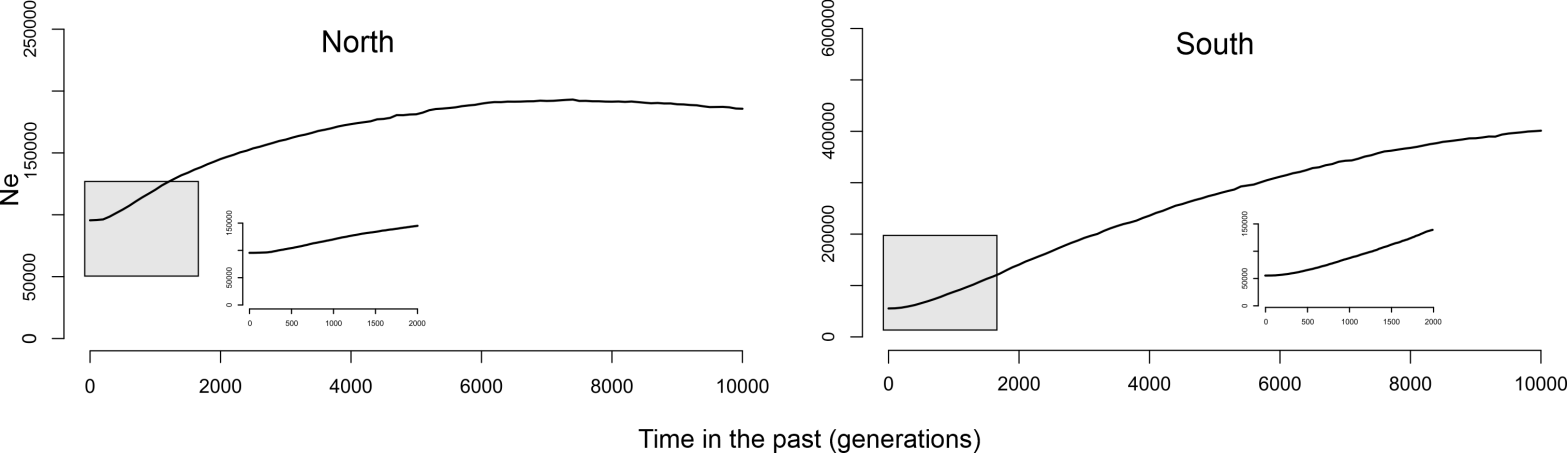
Ne trajectories from present (0) to 10 000 generations ago for two groups of populations of *A. montensis*, estimated with VarEff. Note that the *y*-axis (Ne) has different scales in each plot. The small insert in each plot shows a detail of the trajectory of Ne in the last 2000 generations.

Following Geneland results, we grouped populations in two sets for the estimation of Ne changes with VarEff: Iguazú and Urugua-í (‘North’) and Cuña Pirú and Osununú (‘South’); the population from Martinez Crovetto was not included in this analysis due to the high frequency of null alleles at two loci. The pilot runs revealed that the value of JMAX and the mutation model had little effect on the results. Also, a stable Ne was inferred in all pilot runs for the time period between 20 000 and 10 000 generations before present (electronic supplementary material, figure S1). Therefore, we run the final analysis using JMAX = 4, under the geometric mutation model and a time since the start of the population of 10 000 generations. VarEff identified stable effective population sizes in northern populations until approximately 5000 generations ago, at which point a steady decline began that finished in a stable period in the last 200 years approximately (figure 3). In the southern populations, the ancestral population size would have been larger than in the north, but the decline started earlier, approximately 8000 generations ago, was more pronounced, and resulted in smaller effective sizes in the south compared to the north (figure 3).

### 3.2. Cytochrome b

The sequences of *A. montensis* presented a maximum of 35 nucleotide differences, and a sequence divergence that varied from 0 to 5.51%. A total of 66 haplotypes were identified from 179 sequences of the cyt-b gene (electronic supplementary material, table S2). The haplotype and nucleotide diversity values were 0.905 and 0.00729, respectively.

**Figure 4.**
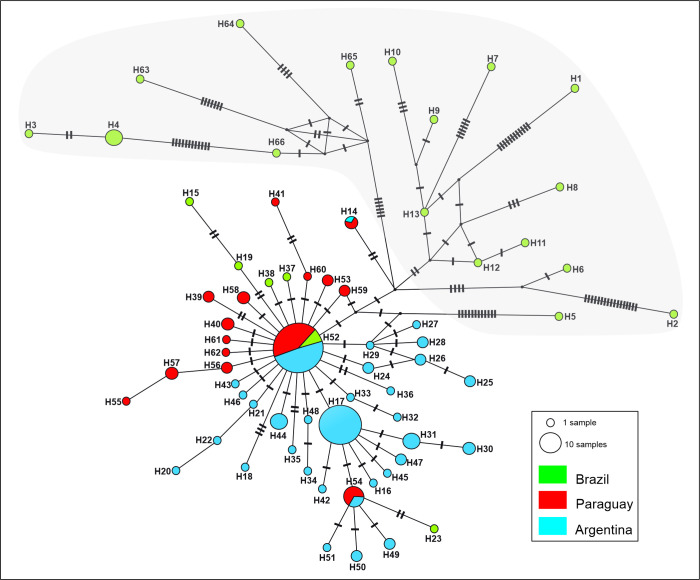
Haplotype network of the cyt-b gene of *Akodon montensis*. The haplotype frequency is represented by circles with different sizes. Colours indicate where the different haplotypes come from: green = Brazil; red = Paraguay; and blue = Argentina. The black segments perpendicular to the lines that connect the haplotypes indicate mutational steps. The missing intermediate haplotypes are represented by black dots. The grey shading area shows divergent haplotypes.

The haplotype network showed a star-like pattern around haplotypes H52 and H17, indicating low levels of divergence between these sequences and their derived, and a high frequency of single mutations (figure 4). Haplotype H52, the most common and widely distributed, was shared by specimens from Paraguay, Brazil and Argentina. Haplotype H17 was shared by numerous specimens from Argentina and is exclusive to this region. Divergent haplotypes, separated by a large number of mutations, were mainly observed in individuals from the northeast of Brazil. For example, the H5 haplotype was recorded in the state of Minas Gerais, separated by 14 mutations from the central H52 haplotype. Haplotypes H9, H10, H63, H64, H65 and H66 were recorded in the Rio de Janeiro state, presenting between 9 and 17 mutations in relation to H52. Haplotypes H1, H2, H6–H8 and H11–H13 were detected in the São Paulo state, and diverge between 5 and 23 mutations from H52. Finally, the H3 and H4 were restricted to the Rio Grande do Sul state, with a difference of 24 and 22 mutations from H52, respectively (figure 4).

**Figure 5.**
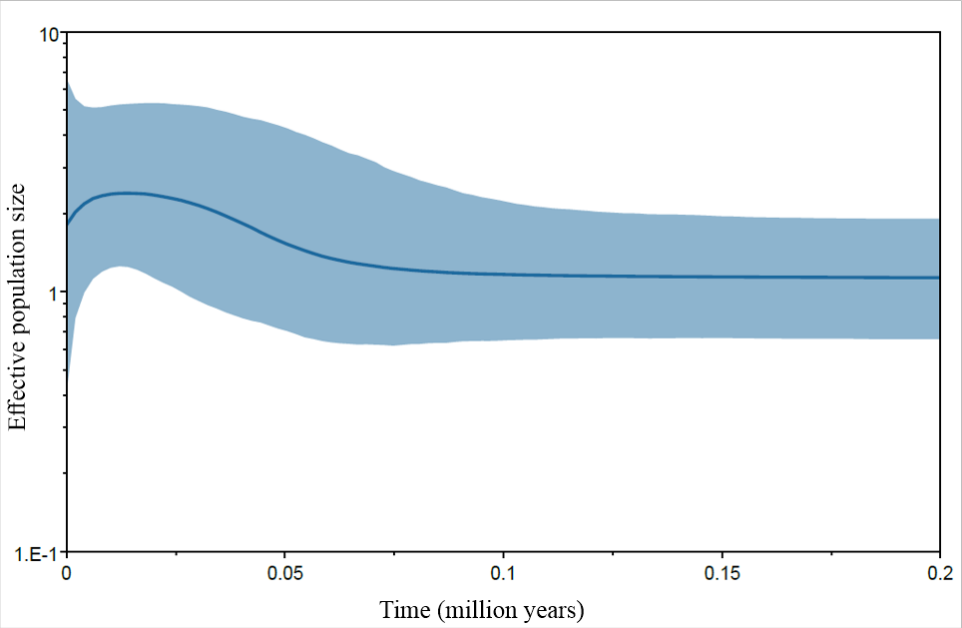
Bayesian skyline plot showing the effective population size (Ne, in *y*-axis) fluctuations through time (in millions of years, in *x*-axis). The bold line represents the median population size estimate and the light blue area represents the 95% higher posterior probability.

Neutrality tests Tajima’s *D* and Fu’s Fs showed negative and significant values (*D* = −2.51363, *p* < 0.001; Fs = −63.374, *p* < 0.0001). The unimodal mismatch distribution supports a relatively recent population expansion (electronic supplementary material, figure S2). The BSP analysis indicates that a demographic expansion started at 70 000 years ago, and then about 10 000 years started a decrease (figure 5).

The spatial diffusion analysis (electronic supplementary material, spatial diffusion analyses) showed that *A. montensis* started its dispersal about ~660 kya in the centre of state of São Paulo. First, a local expansion is observed in adjacent sites in this region and subsequently the beginning of an expansion towards the east, in the centre of the state of Rio de Janeiro. About ~590 kya, the species expanded southwards, reaching to the AF of Misiones province, in the northwest of Argentina. From this last point, about ~550 kya, first the species expanded towards the south of Misiones, to what we now know as the Campos and Malezales ecoregion. Then, *A. montensis* expanded both eastwards and westwards, reaching to the state of Santa Catarina in Brazil and Paraguay, about ~510 kya. Approximately in the same time period, a long-distance expansion occurs from São Paulo towards Rio Grande do Sul. Then, a southwest expansion from Misiones towards the Argentine provinces of Chaco and Formosa occurred around ~470 kya. Finally, we observed short-distance expansions at sites adjacent to regions where the species was previously established.

### 3.3. Ecological niche modelling

In *A. montensis*, the highest contributions were represented by temperature seasonality, and precipitation of the wettest month; these variables explained 73% and 12% of the variation, respectively (electronic supplementary material, table S5).

**Figure 6.**
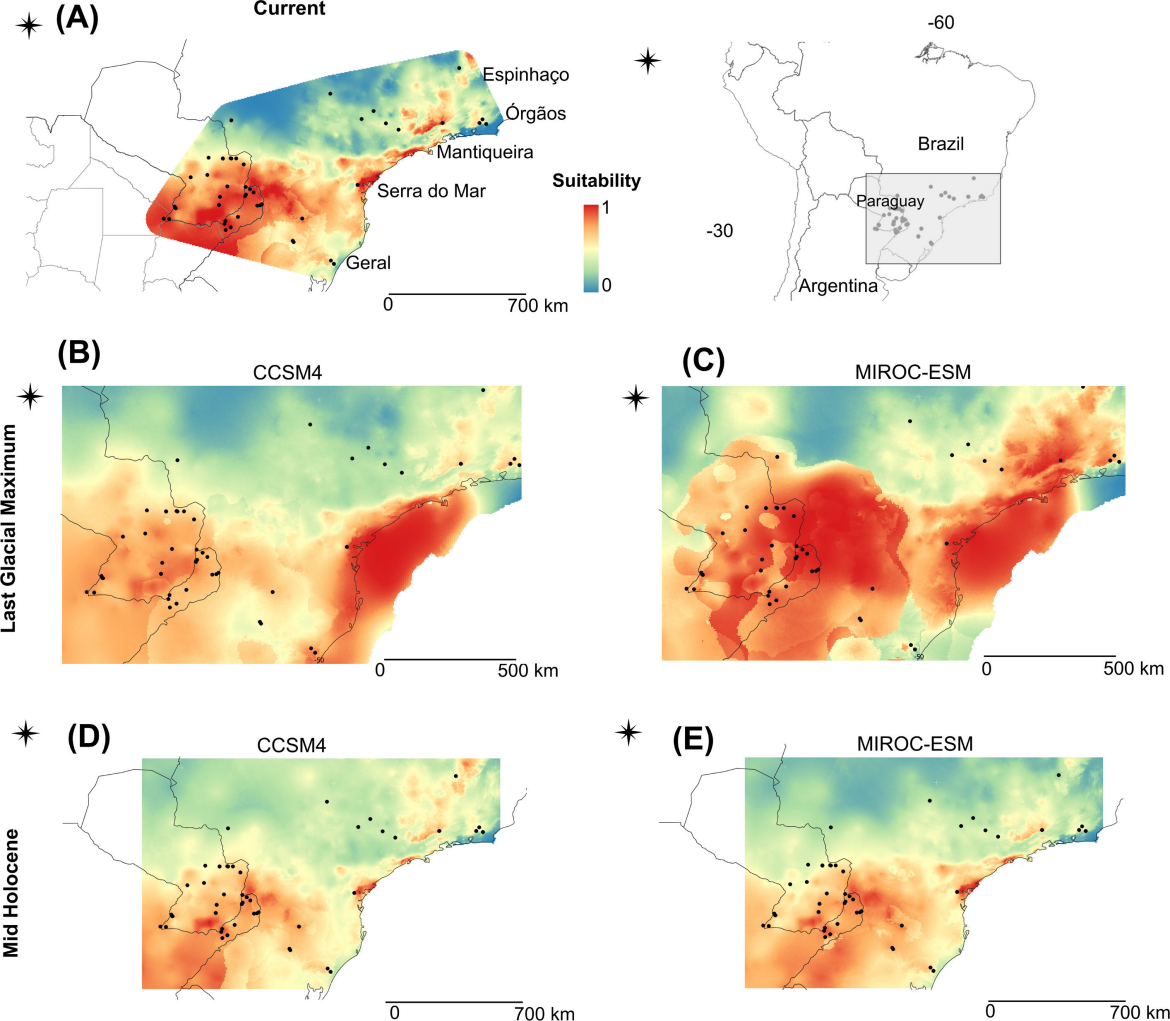
Models of potential distribution for *Akodon montensis* projected on current climate conditions (A) and into two historical scenarios: LGM at 21 Kya (B) and (C) and Mid-Holocene (MH) at 6 Kya (D) and (E). Warmer colours, i.e. red (1 - > 0.75) indicate higher probability occurrence values for each model. Light colours, i.e. white (0.01–0.0) indicate null suitability for the species. CCSM4, Community Climate System Model; MIROC-ESM, Model for Interdisciplinary Research on Climate-Earth System Model.

The estimated current distribution of *A. montensis* is shown in figure 6A. This prediction reveals a continuous area of suitable habitat conditions occurring over a wide area of the southern portion of the AF, which includes southeastern Paraguay, northeastern Argentina, and the border region with Brazil (between ~23–25°S and ~27–29°S). Also, small areas of suitable habitat conditions in southern Brazil on the Atlantic coast along Serra do Mar mountain, and in the interior northern area, were predicted (figure 6A).

Within historical scenarios, both GCMs yielded similar overall results. The projections to the LGM revealed an extensive area of high predicted occurrence of *A. montensis* along the Atlantic coast, including the actual continental shelf (figure 6B,C). The MIROC-ESM model also predicted a large area of suitable habitat along the southeast distribution of *A. montensis*, encompassing southeastern Paraguay, northeastern Argentina and the border region with Brazil (between ~25°S and 29°S; figure 6C), in the CCSM4 model, this area has a lower probability occurrence value. In addition, we observed a favourable small region in the northeast of the distribution of *A. montensis* during the LGM (figure 6C). Furthermore, a small area where *A. montensis* may have occurred during the LGM and at the present time was identified, that corresponds to the Serra Geral mountain region, Rio Grande do Sul state, southeastern Brazil (figure 6A–C).

The projections of MH scenarios revealed a significant reduction in suitable areas for the species compared to the other temporal scenarios. The highest predicted probability (1 - > 0.75) was along the southern part of the distribution, including eastern Paraguay, northeastern Argentina and southern Brazil, between ~25°S and 27°S (figure 6D,E). The favourable region of the east coast of Brazil is predicted to be extremely reduced at this time. The suitable interior region of the north distribution was also reduced, and has lower probability in both models (figure 6D,E).

## 4. Discussion

In this study, we analysed the genetic diversity of *A. montensis* at different spatial scales (locally and across its wide geographic range in the AF) and using different molecular markers (microsatellites and cyt-b, respectively), which retain signatures of demographic events over different temporal scales. This approach allowed us to investigate how historical processes have impacted on its populations. This species is widely distributed in the AF, and its southern distribution limit extends to northern Argentina, mainly in Misiones province. This represents an interesting feature of this study model, as it suggests that, as proposed in previous studies [58], under environmental changes, populations at the edges of a species’ distribution are the most susceptible to being affected. Our population genetics study using microsatellites loci encompasses one of these areas*,* contributing to our understanding of its recent evolutionary history.

In general, the populations of *A. montensis* studied here using microsatellites exhibit high levels of diversity and moderate genetic differentiation among them (Dest estimates). In the south of Misiones, the population of Martínez Crovetto (point 38 in figure 1A) showed the lowest value of Ho. This decrease in heterozygosity could be attributable to the limited sample size of this population (*n* = 7) and, as explained above, to the presence of null alleles in high frequency in two of the four alleles, inasmuch frequencies above 0.20 can underestimate genetic variability within populations and overestimate differentiation between them [59].

Populations of the north of Misiones (points 30 and 31 in figure 1A) exhibited high genetic diversity and low levels of genetic differentiation (Dest estimates). Furthermore, Bayesian clustering analysis grouped these two populations in a single genetic cluster, supporting that in this area there are large populations with high levels of gene flow, in the two protected areas with the largest surface in Misiones, Parque Nacional Iguazú and Parque Provincial Urugua-í. Therefore, these protected areas could be playing an important role in maintaining the genetic variability observed in *A. montensis*. A similar pattern was described for other species, such as the black-and-gold howler monkey *Alouatta carayá* that inhabit Misiones, which seems to maintain gene flow via dispersion through a well-preserved forest, especially in the north of the province [60].

The populations of the centre-south of Misiones showed a moderate genetic differentiation between them (Dest estimates). The fragmentation of the natural habitat increases towards the south of Misiones, driven by contemporary anthropogenic disturbance [6]. Furthermore, the Campos y Malezales ecoregion extends through this area and interdigitates with the Alto Paraná Atlantic Forest, generating a transition zone between these two ecoregions, disrupting the continuity of the AF [61]. In this scenario, it is likely that the southernmost populations of *A. montensis* may be semi-isolated with smaller effective population sizes than those in the north, as showed by VarEff result, leading to a potential decrease in gene flow and genetic variability. Thus, it is probable that multiple factors contributed to generate the genetic pattern observed in the microsatellite markers; assessments with more loci are necessary to further evaluate the differentiation and the gene flow among populations. This becomes particularly important considering the epidemiological significance of this species as a natural host of Jaborá and Ape Aime hantaviruses [20,62]. The results presented here suggest that an outbreak of this disease could spread rapidly in the north of Misiones, Argentina. Although the southern region is more densely populated, the northern area receives a frequent influx of international travellers who may be exposed to the disease, especially given that the most important attraction is the Parque Nacional Iguazú, where this host species inhabits.

Studies of molecular diversity and genetic structure using cyt-b, together with ecological niche modelling and spatial diffusion analysis, taking into account a wide geographic scale covering most of the known geographical range of the species, allowed us to elucidate the past evolutionary history of *A. montensis*. The values of intraspecific genetic distances in cyt-b obtained here are high when compared to those obtained for other species of mammals, including South American rodents, which are in the approximate range of 0–3% [63]. Nevertheless, there are a limited number of studies that include large sample sizes within a single species. Values similar to those obtained for *A. montensis* have been suggested as indicators of the presence of cryptic lineage diversity [63]. However, the continuity of the range of genetic distances, without a clear cut throughout our sample, suggests that *A. montensi*s corresponds to a single species. Nucleotide and haplotype diversity (Pi = 0.00729; Hd = 0.905) obtained in this work exhibited lower values compared to those previously reported for this species (Pi = 0.011; Hd = 1) [14] and for other sigmodontine rodents with a similar distribution in the AF, such as *Akodon cursor* (Hd = 1) and *Euryoryzomys russatus* (Pi = 0.0166; Hd = 0.985) [15,64]. This difference can be explained by two reasons: first, the sequences used in this study are shorter (bp) compared to those used in the mentioned studies; and second, the presence of a high number of similar haplotypes due to population expansions in specific regions.

In a previous phylogeographic work, it has been proposed that the pattern of distribution of genetic variability and the population history of *A. montensis* are concordant with the Carnaval–Moritz refugia model [14]. The authors propose that this species persisted in the São Paulo refugium when climatic conditions were critical, and then colonized from there the interior AF areas, such as northeastern Argentina and eastern Paraguay. However, the niche modelling and genetic data obtained here for *A. montensis* are not consistent with these predictions. In this study, we observed a large area of high habitat suitability on the Brazilian coast during the LGM, known as the Serra do Mar mountain range, and not in the region of São Paulo refugium, which we would expect if *A. montensis* had remained ‘refugeed’ there. Our data suggest that *A. montensis* persisted in Serra do Mar region during the cooler and drier conditions of the LGM, supporting the Atlantis Forest hypothesis [17]. In accordance with this, both GCMs suggest that forested habitats expanded onto the continental shelf during the LGM, resulting in extensive areas of high environmental suitability for *A. montensis*. Additionally, contrary to the Carnaval–Moritz model, demographic history analysis in *A. montensis* indicated a population expansion (Tajima’s *D*, Fu’s Fs and mismatch distribution) that started long before the LGM (BSP analysis), and not after as the model predicts. Similar patterns have been found in other AF rodents, such as *A. cursor* and *E. russatus*, that experimented a population expansion rather than bottlenecks in the same region during the LGM [15,65]. It is important to note that, in the MH and at the current time, this area suffered a significant reduction mainly due to oceanic submersion after deglaciation. This process could have caused the migration of individuals from contiguous flooded areas, which can explain the high genetic diversity currently found in this region.

Moreover, a small suitable area was identified in Brazil, both in the LGM and in the current time: the mountainous region called Serra Geral, where divergent haplotypes are found [14, this work]. According to diffusion analyses, the species colonized this region approximately ~550 kya, which was probably sufficient time for the observed divergence to have occurred. Species distribution models recovered a similar small suitable area for *E. russatus*, a sigmodontine rodent with a similar distribution to *A. montensis*, but it is a more forest-specialist species [15]. Moreover, Serra Geral has previously been identified as a probable refuge for *A. montensis* called ‘Rio Grande do Sul (RGS) refugium’ [14]. The data suggest that in these small regions, *A. montensis* could have persisted during the LGM until the present, potentially playing a role in the diversification of the species.

Our ecological niche models identified an additional area that had not been previously recognized as a highly suitable zone in the potential distribution of *A. montensis*. This area encompasses northeastern Argentina, eastern Paraguay and southwestern Brazil, and persists during the three analysed periods: LGM, MH and the present. According to the diffusion analyses, the northeastern part of Argentina was colonized first, and from there the species expanded and colonized close geographic areas in Paraguay and Brazil, before the LGM. This finding suggests a high probability of occurrence of the species in this region at different chronological periods, even during less favourable environmental conditions as in the MH, when the extent of suitable habitat for the species was markedly reduced. In the LGM, the niche models suggest an increase in the geographic range of this species, especially to the east, but also to the west. This is congruent with a population expansion dating before the LGM. It is noteworthy that in current times, northeastern Argentina, particularly the Misiones province, conserves a substantially continuous portion of AF through a system of natural reserves (figure 1B) that has been identified as an area of endemism for several taxonomic groups [6,66]. The increase of the area with favourable environmental conditions in this region could explain the high population density of *A. montensis*, as well as the presence of other rodent species such as *E. russatus* with more specialized ecological requirements [15].

The spatiotemporal patterns underscore the central role of climatic variables in shaping the ecological niche of *A. montensis*. Specifically, the ecological niche model revealed a pronounced influence of temperature and precipitation-related variables, with temperature seasonality emerging as particularly significant. This variable, alongside precipitation of the wettest month, could indicate that the species’ current distribution is primarily shaped by thermal fluctuations and seasonal rainfall peaks, suggesting that the species may be particularly sensitive to seasonal climatic trends. Notably, variables such as annual mean temperature, precipitation of the driest month and temperature of the wettest quarter showed minimal or negligible contributions to the model. This low association could suggest that *A. montensis* may tolerate a relatively broad range of background climatic conditions. Model performance metrics (e.g. AUC or AICc) also indicated moderate predictive ability, which is expected for species with relatively broad ecological tolerances [67]. Overall, these results support the idea that the species’ range is shaped more by seasonality than by extremes or averages, which may have important implications when interpreting its historical responses to glacial times.

Several studies, aimed at inferring the historical and contemporary processes shaping AF species populations, have indicated that multiple factors have influenced this biota simultaneously over time, with impacts varying across regions and species [10,12,13,65,68]. The responses of each species to environmental changes were largely determined by life-history traits, such as thermal tolerance, dispersal ability, abundance and resource specialization (e.g. generalists versus specialists) [8,69]. Additionally, adjacent and ecologically connected biomes have served as important sources of biological diversity throughout the climatic oscillations experienced by the AF [70–72]. During the LGM, signals of population expansion in several unrelated taxa—including insects, birds and mammals—support an expansion into the eastern coastal region of this biome, consistent with the Atlantis Forest hypothesis [65,72–74, this work]. At that time, the significant changes in land distribution caused by sea level lowering likely had major impacts on the continental adjacent biota. Floristic reconstructions suggest a grassland–forest mosaic across the exposed continental shelf during the LGM, followed by substantial shifts in the AF community composition in the post-glacial period [68]. After the LGM, the geographic range of the AF contracted, and areas of high habitat suitability for many species along Brazil´s eastern coast persisted only in a narrow belt. This is in accordance with the population decline observed in some taxa, such as *A. montensis* and *A. cursor*, in more recent times [65, this work]. Currently, anthropogenic activities have drastically altered the AF in Brazil, further reducing the availability of suitable habitat for forest-dependent species, many of which currently exhibit broader suitable areas in the AF of Argentina and Paraguay [17,74, this work]. Our study contributes to understanding these historical processes by revealing a complex evolutionary scenario for *A. montensis*, characterized by distinct demographic histories across its distribution. These findings underscore the importance of integrating multiple genetic loci with ecological data to gain a more comprehensive understanding of the natural history of this highly diverse and deeply endangered biome.

## Data Availability

Supplementary material is available online [75].

## Authors' Notes

In author contributions D.A.R.D.O. A.N.D.R.E.A.M.A.R.T.I corresponds to DAM
